# Characterization of the Spoilage Microbiota of Hake Fillets Packaged Under a Modified Atmosphere (MAP) Rich in CO_2_ (50% CO_2_/50% N_2_) and Stored at Different Temperatures

**DOI:** 10.3390/foods8100489

**Published:** 2019-10-13

**Authors:** Adriana Antunes-Rohling, Silvia Calero, Nabil Halaihel, Pedro Marquina, Javier Raso, Juan Calanche, José Antonio Beltrán, Ignacio Álvarez, Guillermo Cebrián

**Affiliations:** 1 Departamento de Producción Animal y Ciencia de los Alimentos, Facultad de Veterinaria, Instituto Agroalimentario de Aragón– IA2, Universidad de Zaragoza-CITA, 50013 Zaragoza, Spain; dri.rohling@gmail.com (A.A.-R.); jraso@unizar.es (J.R.); calanche@unizar.es (J.C.); jbeltran@unizar.es (J.A.B.); ialvalan@unizar.es (I.Á.); 2 Departamento I+D+i, Alquizvetek S.L, Zaragoza, 50013 Zaragoza, Spain; silviacalero@alquizvetek.com (S.C.);; 3 Departamento de Patología Animal, Facultad de Veterinaria, Universidad de Zaragoza, 50013 Zaragoza, Spain

**Keywords:** fish, *Photobacterium*, shelf-life, high-throughput gene sequencing

## Abstract

The aim of this study was to characterize the spoilage microbiota of hake fillets stored under modified atmospheres (MAP) (50% CO_2_/50% N_2_) at different temperatures using high-throughput 16S rRNA gene sequencing and to compare the results with those obtained using traditional microbiology techniques. The results obtained indicate that, as expected, higher storage temperatures lead to shorter shelf-lives (the time of sensory rejection by panelists). Thus, the shelf-life decreased from six days to two days for Batch A when the storage temperature increased from 1 to 7 °C, and from five to two days—when the same increase in storage temperature was compared—for Batch B. In all cases, the trimethylamine (TMA) levels measured at the time of sensory rejection of hake fillets exceeded the recommended threshold of 5 mg/100 g. *Photobacterium* and *Psychrobacter* were the most abundant genera at the time of spoilage in all but one of the samples analyzed: Thus, *Photobacterium* represented between 19% and 46%, and *Psychrobacter* between 27% and 38% of the total microbiota. They were followed by *Moritella*, *Carnobacterium,* *Shewanella,* and *Vibrio*, whose relative order varied depending on the sample/batch analyzed. These results highlight the relevance of *Photobacterium* as a spoiler of hake stored in atmospheres rich in CO_2_. Further research will be required to elucidate if other microorganisms, such as *Psychrobacter*, *Moritella,* or *Carnobacterium*, also contribute to spoilage of hake when stored under MAP.

## 1. Introduction

The term ‘hake’ mainly refers to fish of the genera *Merluccius*, although other cod-like fish are also known as hake [1]. This genus is present around the globe and makes up a considerable proportion of the worldwide catch of fish. In 2014, 1.3 million tons of hake were caught, representing nearly 1.5% of all catches worldwide [2]. The genus *Merluccius* includes several species. Among them, the one caught in the Mediterranean and North East Atlantic area is European hake: *Merluccius merluccius*. This species is of particular relevance for the fishing sector in Spain [3]. Thus, in 2014, around 120,000 tons of European hake were caught in Spain, which represents approximately 60% of the total annual catches worldwide of this species [4]. 

In the Mediterranean region, hake has traditionally been stored in ice during transport and consumed fresh [3], but the amount of commercialized hake packaged under modified atmospheres (MAP) is quickly rising, mainly due to market globalization and changes in purchasing habits, along with the resulting need for an increase in shelf-life that they impose [5,6]. Nevertheless, in order to apply this preservation technology efficiently—and to further improve it—it is necessary to be able to precisely identify the agents responsible for the spoilage of fish stored under MAP conditions, along with the sensorial and physico-chemical changes which they induce, and which will ultimately lead to consumer rejection of the product. 

Until recently, most studies dealing with food spoilage and the identification of the microorganisms responsible for it have been based on conventional microbiology, i.e., culture-based methods. However, these studies often did not provide a complete overview of the entire microbial diversity in complex environments [7], such as packed fish. High-throughput sequencing technology has become a widely used tool for the study of whole communities of prokaryotes in many niches [8], including fish stored under different conditions [9,10,11,12,13,14]. Nevertheless, this technique also has its own perils and pitfalls, most of which have already been summarized by Janda and Abbott [15], Ercolini [16] and, more recently, by Rodrigues et al. [17].

The aim of this study was to characterize the spoilage microbiota of hake fillets stored under MAP (50% CO_2_/50% N_2_) at different temperatures using high-throughput 16S rRNA gene sequencing and to compare these results with those obtained using traditional microbiology techniques.

## 2. Material and Methods

### 2.1. Raw Material, Modified Atmosphere Packaging, Storage Conditions, and Experimental Design

Fresh hake fillets with skin (*Merluccius merluccius*, captured at the Bay of Biscay, FAO zone 27.8), supplied by Skanfisk S.L., were packaged (500 ± 10 g per package) under a modified atmosphere (50% CO_2_/50% N_2_) with a gas/product ratio of approximately 2:1 and then stored at different temperatures (1, 4, and 7 °C). Temperature and gas composition of the headspace of the packages were measured along the shelf-life study by means of a thermocouple data logger (Lascar, Whiteparish, UK), which recorded the temperature throughout the study, and by means of a headspace gas analyzer (Oxybaby GmbH & Co, Witten, Germany) used to measure the headspace of packages at each sampling time. 

Two different batches (A and B) were studied in different work periods (March and June 2018). Each batch was divided into three sub-batches, and each sub-batch was subsequently stored at each of the three pre-defined temperatures (1, 4, and 7 °C) for further analysis. Six packages were selected and analyzed on each sampling day. Three of the packages were used for sensory and biochemical analysis, and another three, for standard microbiological analysis and DNA purification. 

### 2.2. Sensory and Biochemical Analysis 

A panel of twelve selected assessors with previous experience in sensory analysis of fish freshness was chosen from the staff of Faculty of Veterinary of the Universidad de Zaragoza. The panel received prior training on the use of QIM assessment scoresheets to evaluate different attributes in fish according to requirements of ISO standards (ISO 8586: 2012 [18]). The panel performance was checked following the guidelines established in Section 8, titled “Analysis of Results” of the ISO standard 8556:2012 (ISO 8586: 2012 [18]), including one-way ANOVA analysis for establishing the discriminatory capacity of the panel and three-way ANOVA (products, assessors, and session) to evaluate the reproducibility. The evaluation of individual performance was carried out by determining the individual coefficients of variation (CV) for each assessor.

Sensory evaluations were carried out using the quality index method (QIM) scheme for white fish fillet [19] that was adapted to hake fillets. The QIM score was based on the appearance, colour, texture, and odour of raw hake fillets (Table 1). The QIM score was the sum of the scores given by the sensory panel on individual quality parameters on a scale from zero to 14 (the higher the value, the worse the freshness of the fish). Samples with a QIM score higher than 6.5 were considered as spoiled.

Trimethylamine (TMA) determination was carried out using the picric acid method as described in Woyewoda et al. [20]. Results were reported as the average of two replicates per sample.

### 2.3. Standard Microbiological Analysis

A 25 g portion from each package was aseptically weighed and mixed with 225 mL of previously sterilized buffered peptone water (BPW, Oxoid, Hamphsire, UK) in a sterile plastic bag, and blended with a Stomacher 400 Circulator (Seward, Worthing, UK) for 30 s. The bacterial groups were enumerated as described in Table 2, except for *Photobacterium phosphoreum*, which was enumerated using qPCR, as described in Antunes et al. [21].

### 2.4. Bacterial DNA Extraction from Hake Fillets 

A Maxwell 16 Lev Blood DNA Extraction Kit (Promega, Madison, USA) was used to purify bacterial DNA from hake samples (BPW homogenate, as described above), according to the manufacturer’s instructions. The extracted DNA was then used to perform qPCR analysis of *P. phosphoreum*, *Shewanella* spp., and *Pseudomonas* spp. and/or for high-throughput 16S rRNA gene sequencing analysis. 

For qPCR assays, DNA was extracted from 0.2 mL of BPW homogenate of each sample and then analyzed separately, as described below.

For high-throughput 16S rRNA gene sequencing analysis, 16 mL of homogenate of each of the three samples were concentrated by centrifugation (10,000 g/5 min). It was checked via qPCR analysis that concentrating the homogenates did not exert an influence on the results obtained (data not shown). The three samples were subsequently pooled before extraction. High-throughput 16S rRNA gene sequencing analysis was carried out as described below. 

### 2.5. qPCR Assays

Real-time PCR amplification was performed using the GoTaq qPCR Master Mix (Promega, Madison, USA) and the primers indicated in Table 3. The qPCR assays were carried out with a CFX connect real-time system (Bio-Rad Laboratories, Hercules, USA) following a 5-min protocol at 94 °C for GoTaq enzyme activation, followed by 44 cycles of 94 °C for 10 and 40 s at a temperature of 55 °C for annealing, elongation, and fluorescence data acquisition. A melting curve between 65 and 90 °C was obtained after the last amplification cycle, and at a temperature transition rate of 0.5 °C/s. All amplification reactions were run in triplicate.

### 2.6. High-Throughput 16S rRNA Gene Sequencing

PCR libraries were prepared by targeting the V3–V4 hypervariable regions of the 16S rRNA (Fw primer: TCGTCGGCAGCGTCAGATGTGTATAAGAGACAGCCTACGGGNGGCWGCAG; Rev primer: GTCTCGTGGGCTCGGAGATGTGTATAAGAGACAGGACTACHVGGGTATCTAATCC) [23] using the Illumina 16S Metagenomic Sequencing Library preparation protocol. The generated DNA libraries were sequenced with MiSeq Reagent Kit v3 in the lllumina MiSeq platform, using 300 bp paired-end sequencing reads. Sequenced samples were then processed with QIIME2 v2018.6.0 [24] and the DADA2 plug-in [25]. The resulting sequences were clustered in amplicon sequence variants (ASVs) and then classified by taxon using a fitted classifier. The scikit-learn classifier was used to train the classifier using the SILVA (release 132 QIIME) database, with a clustering threshold of 97% similarity. For classification purposes, only ASVs containing at least 10 sequence reads were considered significant. Sequences were deposited in the NCBI Sequence Read Archive (BioProject: PRJNA574112).

### 2.7. Statistical Analysis

GraphPad PRISM software (Graph Software, San Diego, CA, USA) was used for statistical analyses (analysis of variance and student’s *t*-test) (*p* = 0.05).

## 3. Results

### 3.1. Determination of the Time of Spoilage through Sensory and Biochemical Analysis 

The time at which hake fillets were considered as spoiled was determined by sensory analysis using the QIM method, as described in Materials and Methods. That time corresponded with Day six, three, and two for the hake fillets of Batch A stored at 1, 4, and 7 °C, respectively, and with Day five, five, and three, for the hake fillets of Batch B stored at 1, 4, and 7 °C, respectively. According to the obtained results, the major causes of rejection of hake fillets by the panelists would be the appearance of unpleasant and offensive off-odours and the changes in aspect/colour of the skin (which turned whitish and lost brightness). These were the only two attributes whose scores increased progressively and significantly (*p* < 0.05) throughout storage for all the studied samples (for the two batches and at the three studied temperatures). 

**Figure 1:**
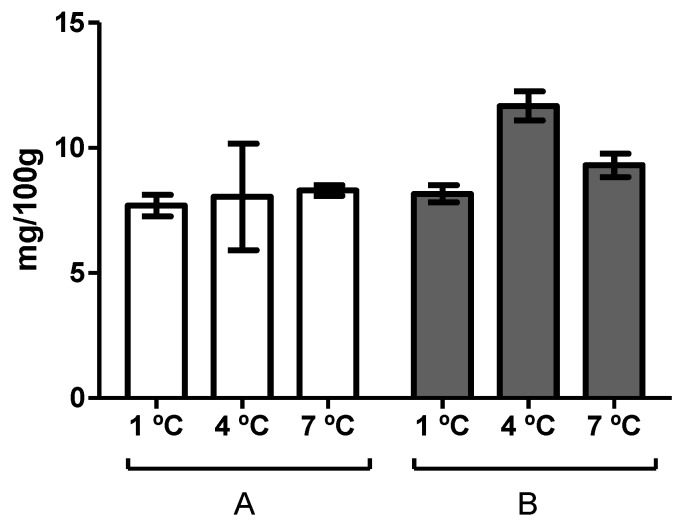
Trimethylamine (TMA) content at the time of spoilage of hake fillets stored in modified atmosphere (MAP) of the two batches studied (A and B) and at each of the storage temperatures investigated (1, 4, and 7 °C). Error bars represent the standard deviation.

On the other hand, Figure 1 shows the trimethylamine (TMA) values determined at the day of spoilage of the hake fillets stored at different temperatures, as determined by sensory analysis. As can be observed, these values were higher than 5 mg-N TMA 100 g−1—which is the limit of acceptability proposed by Baixas-Nogueiras et al. [26] for hake—for all the samples. It should be noted that the time of spoilage determined via sensory analysis was coincident with the day on which samples surpassed the threshold limit indicated above for batch A, but not for batch B, for which it was surpassed 1–2 days before (data not shown).

### 3.2. qPCR Analysis

As described above high-throughput 16S rRNA gene sequencing analysis was carried out with pooled samples. However, this process (pooling) might lead to biased results, since samples with high microbial counts will be overrepresented in the libraries. Thus, in parallel with the high-throughput 16S rRNA gene sequencing, the amount of *P. phosphoreum*, *Shewanella* spp., and *Pseudomonas* spp., of each of the three independent samples to be pooled was analyzed with qPCR. Table 4 shows the results obtained for the first batch studied. Similar results were obtained for Batch B. As can be observed in the table, the differences in Cq (quantitation cycle) among the packages corresponding to the same sampling point (biological replicates) were only greater than 2.5 Cq in the case of one sample (*Pseudomonas* spp.; 1 °C). From these results, it can also be concluded that variance among biological replicates is comparable to that observed between technical replicates (amplification replicates of the same sample). Thus, standard deviations among technical replicates varied from 0.09 to 2.14 Cq, and standard deviations among biological replicates varied between 0.18 and 2.79 Cq. The average of the standard deviations was also very similar: 0.81 for technical replicates and 0.97 for biological replicates. This would indicate that pooling the samples would not result in biased results, at least under our experimental conditions and for the assayed microorganisms.

### 3.3. High-Throughput 16S rRNA Gene Sequencing

**Figure 2:**
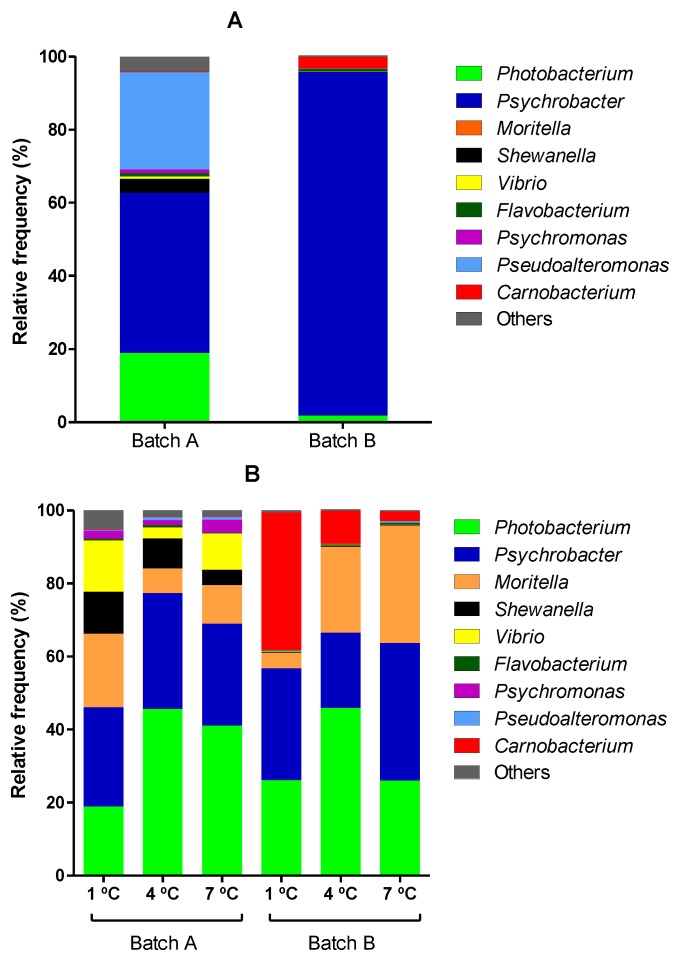
Metagenomic analysis of the composition of the microbiota of hake fillets at day zero (**A**) and at the time of spoilage (**B**) of the two batches studied (Batch A and Batch B) and at each of the storage temperatures investigated (1, 4, and 7 °C).

High-throughput 16S rRNA gene sequencing analysis was applied to determine the composition of the microbiota of the two studied batches of hake fillets stored under MAP at different temperatures. Figure 2 shows the results obtained for the hake fillets at day zero (2A) and at the time of spoilage (2B). 

Figure 2A shows the composition of the microbiota of hake fillets at day zero (before packaging) of the two batches. As can be observed, *Psychrobacter* was the most abundant genus in both batches, but whereas in fillets of Batch A, *Pseudoalteromonas* accounted for up to 26.7%, *Photobacterium* for 18%, *Shewanella* for 3.7%, and *Psychromonas*, *Flavobacterium,* and *Vibrio* for more than 0.5% (0.98%, 0.92%, and 0.66%, respectively), the most abundant genera in fillets of Batch B following *Psychrobacter* (93.9%), were *Carnobacterium* (3.2%), *Photobacterium*, (1.8%), and *Flavobacterium* (0.80%). These differences cannot be attributed to differences in the total initial number of microorganisms (which would suggest different spoilage states) since the counts of both total aerobic and anaerobic psycrotrophs at day zero differed less than 2-fold between the two batches. Further work should be carried out in order to determine the causes of such differences.

On the other hand, as can be observed in Figure 2B, *Photobacterium* and *Psychrobacter* are the dominant genera in hake fillets at the time of spoilage in all analyzed samples but one (Batch B; storage temperature 1 °C). *Photobacterium* represented between 19% and 46% and *Psychrobacter* between 27% and 38% of all microbiota. They were followed by *Moritella*, *Carnobacterium*, *Shewanella*, and *Vibrio*, whose relative order varied depending on the sample/batch analyzed. The relative amount of *Moritella* was high in all samples (ranging from 4.3% to 32.2%) and was the third most abundant microorganism in four out of six samples. However, the amounts of *Shewanella*, *Vibrio*, and *Carnobacterium* were very different depending on the batch and the sample analyzed. Thus, *Shewanella* was much more abundant in spoiled hake samples of the first batch than in those of the second one (7.92% versus 0.30%). Similar results were obtained for *Vibrio*. Conversely, *Carnobacterium* was much more abundant in the spoiled samples of the second batch (0.18% versus 16.4%). Regarding this latter microorganism, considerable differences were also found among samples coming from Batch B depending on temperature: The relative abundance of this microorganism was higher in spoiled samples the lower the temperature assayed.

Comparison of results obtained for hake fillets at day zero and at the time of spoilage leads to several deductions. First of all, a decrease in the relative amount of *Psychrobacter* was observed. Likewise, for Batch A, a marked decrease in the relative count of *Pseudoalteromonas* was observed. By contrast, the relative *Photobacterium* count (in all samples but one, for which the number was equal to 18.9%) and, especially, of *Moritella* (from 0.04% to 16.3% on average) increased. It should also be noted that the data obtained for Day zero fillets might also help to explain the differences between batches at the time of spoilage, since it was observed that, at day zero, *Shewanella* and *Vibrio* were much more abundant (in relative terms) in hake fillets of Batch A and *Carnobacterium* in those of Batch B. 

### 3.4. Standard Microbiological Analysis

**Figure 3:**
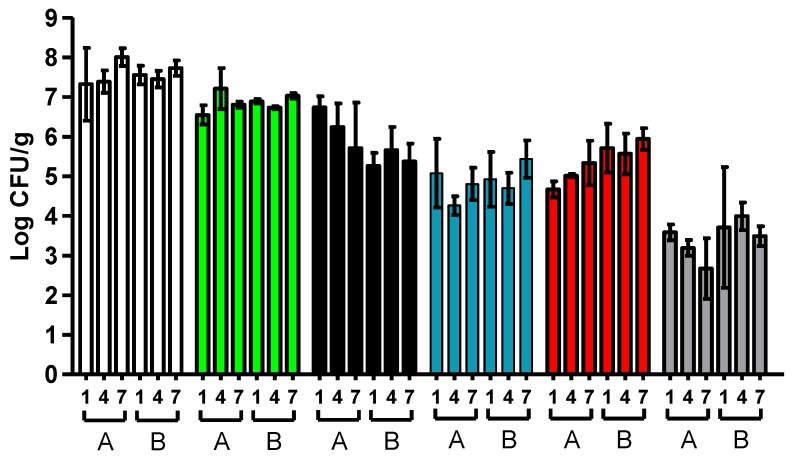
Microbial counts (Log CFU/g) at the time of spoilage of hake fillets stored in MAP of the two batches studied (A and B) and at each of the temperatures investigated (1, 4, and 7 °C). Anaerobic psychrotrophs (white bars), *Photobacterium* (green bars), *Shewanella* (black bars), *Pseudomonas* (blue bars), Lactic acid bacteria (red bars) and *Enterobacteriaceae* (grey bars). Error bars represent the standard deviation.

The concentration of the different microbial groups studied at the time of spoilage (end of shelf-life) of hake fillets was also studied by means of traditional microbiological techniques. The obtained results are shown in Figure 3. At the time of spoilage, total microbial counts (total anaerobic psychrotrophs) were over 7.3 log units for all samples. Among the microbial groups investigated, the most abundant microorganisms were those belonging to the genera *Photobacterium* and *Shewanella*, which reached up to 6.8 and 5.9 log10 cycles—on average—respectively. They were followed by Lactic acid bacteria, Pseudomonas, and Enterobacteriaceae, in that order. Few differences in microbial counts were found among samples analyzed at the time of spoilage, regardless of the storage temperature and the batch studied. Thus, significant differences were only found between the counts of Lactic acid bacteria (*p* < 0.05) corresponding to the sample stored at 1 °C from the first batch and the sample stored at 7 °C from the second batch. Nevertheless, it should also be noted that the *Shewanella* counts were lower (in general) in spoiled hake samples of the second batch.

## 4. Discussion

In this investigation, we studied the spoilage microbiota of hake fillets stored under MAP (50% CO_2_/50% N_2_) at different temperatures. To the best of our knowledge, this is the first study to describe the spoilage microbiota of hake stored under MAP via high-throughput 16s rRNA gene sequencing. The time of spoilage was determined through sensorial and TMA analysis and, once determined, microbiological analyses were carried out. 

As observed in our study, one of the most common causes of sensory rejection of fish and seafood can be found in unpleasant and offensive off-odours [27,28]. The volatile compounds responsible for these off-odours include alcohols, ketones, sulphur compounds, amines (trimethylamine [TMA], dimethylamine [DMA]), esters, aldehydes, and organic acids that result from bacterial degradation of soluble, low-molecular-weight components [29,30]. However, not all the bacteria that make up the microbiota of fish are capable of generating these volatile compounds, and those that are capable thereof are generally referred to as “specific spoilage organisms” or SSOs [5]. It should be noted that, in many cases, SSOs do not dominate the microbiota of fresh fish; however, due to their ability to grow faster than their competitors under particular storage conditions (temperature, atmosphere, and others) they become the dominant spoilage microbiota. Thus, the SSO responsible for the spoilage of a particular fish product would mainly depend on the initial microbiota of that product (which will also depend on fish species, geographical origin, previous processing conditions, etc.), and on storage conditions [29]. 

Regarding the techniques used in this study to characterize the microbiota at the time of spoilage of hake fillets stored under MAP, it should be noted that, as described in Hilton et al. [31], cultivation-based methods remain the most widely used methods due to their extensive validation and their cost-effectiveness. They also enable an estimation of the total number of viable bacteria (and of certain bacterial groups) with reasonable precision (standard deviation of 20% for three technical replicates) (estimated standard deviation for the results of three replicate samples > 10%; [32]). However, due to the limitations of the media utilized for growth, an inherent bias to cultures will exist. Therefore, culture techniques may not be effective at identifying the presence of unknown or known but unculturable microorganisms [33]. Additionally, in many cases, microbial identification requires additional biochemical tests. PCR assays (as the one used here for quantifying *Photobacterium*) have many advantages as described elsewhere [34] but, as for culture methods, one of the weaknesses of PCR is that target sequences for primer design must be chosen before testing begins. By contrast, HTS is the most in-depth and unbiased method of obtaining genomic or metagenomic information [17,35]. Unlike PCR or microarrays, it does not require primer or probe design, it can be easily multiplexed, and the specificity and selectivity of the sequencing can be adjusted computationally after acquiring the data [36,37]. High-throughput 16S rRNA gene sequencing is the most widely used technique for microbial diversity analysis and has been applied to various environments [31], including seafood [17]. It nevertheless has some pitfalls, mainly its limitation to differentiate at the species level as well as between live and dead microorganisms; furthermore, it cannot be used to determine the absolute number of microorganisms in a sample [15,16,17]. 

It follows that each technique has its own advantages and pitfalls. These techniques should, therefore, be regarded as complementary and not exclusive. The combination thereof in this study has enabled us to determine the total microbial concentration at the time of spoilage and, since a good correspondence was among the two techniques (data not shown), the number of microorganisms of each bacterial group can be roughly estimated. In any case, it should be pointed out that the relative microbial abundance, as determined by traditional microbiology techniques, should only be taken as an estimation since the media used for enumerating the different microbial groups are not fully selective or differential. 

Regarding the initial microbiota of hake fillets, all the genera identified here as predominant are among those generally reported in the literature, and more specifically, among those found in fish coming from cold or temperate waters [12,14,38], which can be expected in view of our samples’ geographic origin (FAO zone 27.8). Other relevant findings of our investigation that deserve further study are the extended differences in microbial composition among the two batches studied, and the relatively high proportion of microorganisms observed in the genus *Photobacterium* (especially, in Batch A). As previously pointed out, these differences between batches cannot be attributed to differences in the initial microbial load, but the high numbers of *Photobacterium* might be attributed to the fact that the initial microbial load was quite high (≈ 1–2 × 10^5^ CFU/g). This high initial microbial load is comparable to the load reported in previous works [39], yet very close to the upper boundary. In this sense, although *Photobacterium* is considered as a typical SSO of fish stored under atmospheres containing high levels of CO_2_, as will be discussed below, the results of Reynisson et al. [40] indicate that it can also grow very fast under aerobic conditions and it should also be remarked that Kuuliala et al. [12] already observed a high proportion of *Photobacterium* in fresh cod in some of the batches they studied, which might explain the results obtained here. 

At the time of spoilage, *Photobacterium* and *Psychrobacter* dominated the microbiota of spoiled hake fillets stored under MAP in all but one of the studied conditions. This finding is similar to that reported by Kuuliala et al. [14] for cod stored under certain MAP conditions similar to ours (60% CO_2_/5% O_2_/35% N_2_). Although in their case, they also found a high proportion of *Acinetobacter* and *Brochothrix*. *Photobacterium* is generally regarded as the major SSO of cod stored under MAP (when it includes CO_2_) [23] and one of the most relevant in coalfish, halibut, salmon, and hake, among other fish species, [21,39,41,42,43] stored under the same conditions. Given the high amount of *Photobacterium* found within the microbiota at the time of spoilage for both batches and at all temperatures tested, the fact that off-odours were the major cause of rejection by the panelists and that, since, according to Sahidi et al. [44], the index of trimethylamine (TMA) production per CFU (TMA/CFU) of *Photobacterium phosphoreum* would be 30 times higher than that of *Shewanella putrefaciens*, and much higher than that of other SSOs, our results strongly suggest that, as for cod, the major SSO of hake (another *Gadidae*) stored under MAP (50% CO_2_/50% N_2_) is *Photobacterium*. Furthermore, according to our data, this would apply regardless of storage temperature (1–7 °C) and the initial composition of the microbiota of hake fillets.

On the other hand, *Psychrobacter* species belong to the group of spoilage microbiota found on chilled proteinaceous foods stored in air [10,12,13,45]. Our results indicate that this genus would represent between 20.5% and 37.6% of all microbiota at the time of spoilage of hake fillets stored under MAP. Its abundant presence has also been observed on spoiled cod and other spoiled seafood products [22,42,46,47,48]. However, the species of this genus have been considered to be moderate spoilers, as some produce only weak off-flavours or slightly fishy, musty off-odours [49,50,51]; moreover, this genus lacks important food spoilage attributes such as proteolysis and production of sulphides [52]. Thus, Broekaert et al. [53] observed that the *Psychrobacter* isolates they studied did not produce significant amounts of volatile organic compounds; thus, their contribution to fish spoilage might be marginal. However, those authors also observed that those isolates were able to break down short- to medium-chain (C4–C8) lipids and to hydrolyze amino acids (leucine arylamidase), and that they were able to compete with common spoilage microorganisms—as we have also observed in this investigation—thereby indicating that further study would be required to fully elucidate the role of *Psychrobacter* in fish spoilage.

*Carnobacterium* is a genus of Lactic acid bacteria (LAB) that has been frequently isolated from cold and temperate environments; it was the most abundant (in relative terms) genus under one of the studied conditions (Batch B; storage temperature: 1 °C). This genus consists of nine species, but only two of them, *C. divergens* and *C. maltaromaticum*, are frequently encountered in the environment and in foods [10,12,13,14,54]. It has been reported that these species are the most prevalent in the microbial communities of modified atmosphere-packed (MAP) coalfish, cod, pollack, rainbow trout, salmon, shrimp, swordfish, and tuna, although it seems that they acquire particular relevance if the fish products have been previously frozen [54]. The latter process would lead to the inactivation of *P. phosphoreum*, which might offer some explanation as to why *Carnobacterium* would dominate the spoilage microbiota of these products. Regarding the role of the latter as a potential hake spoiler, it seems that other members of the bacterial community are typically more important in terms of their sensory effect [54]. In any case, it should once more be noted that, whereas *Carnobacterium* represented up to 37% of the total microbiota in Batch B, it accounted for less than 0.3% in Batch A. Since the shelf-life of both batches and the causes of rejection by the panelists were quite similar, this strongly suggests that the contribution of *Carnobacterium* to hake spoilage under the conditions studied in this paper would be very limited. Furthermore, although it has been reported that spoilage was enhanced if moderate-spoilage strains of *C. maltaromaticum* were inoculated with non-spoilage *Vibrio* sp. strains or indeed, the moderate-spoilage organism *Brochothrix thermosphacta* into cold-smoked salmon that was subsequently vacuum-packaged, this was not the case when combined with *P. phosphoreum* [55], which seems to be the major SSO of hake under our experimental conditions. In addition, it should be noted that results obtained for Batch B suggest that the relative abundance of *Carnobacterium* in hake fillets would be higher the lower the storage temperature. This point nevertheless remains to be corroborated.

As described for *Carnobacterium*, the relative amount of microorganisms of other genera at the time of spoilage also varied widely depending on the batch studied. This was the case of *Shewanella, Vibrio, Psychromonas*, and *Flavobacterium*, some of which are well-known fish spoilers, particularly *Shewanella* [29,38]. For the same reasons as indicated above, it can be speculated that the role of *Shewanella* in the spoilage of hake fillets stored under MAP would be secondary, although it cannot be discarded that it could have contributed to the spoilage of the hake fillets in Batch A. Apart from their value for determining the major SSOs in hake fillets stored under MAP, the results obtained for these five genera (*Carnobacterium*, *Shewanella, Vibrio, Psychromonas*, and *Flavobacterium*) reinforce the assumption that, as would be expected, the composition of the microbiota of fresh fish is a key factor that determines the proportion of microbiota during shelf-life, since a clear relationship between the relative abundance of these five genera in fresh and spoiled fish could be observed in this study when comparing the two batches analyzed. 

*Pseudoalteromonas* is another potential spoiler that has been shown to have high spoilage potential when fish is stored under aerobic conditions [53]. However, in contrast to other studies [22,40,47,48], it accounted for less than 1% of the total microbiota in our study, regardless of batch and storage temperature. Furthermore, its relative abundance decreased markedly when compared to the fresh product. By contrast, the relative abundance of the microorganisms of the genus *Moritella* markedly increased from day zero to the day of spoilage (at least 100-fold in all samples and batches), making this genus one of the most abundant in spoiled hake fillets (the 2nd to 5th most abundant depending on the sample; 3rd in four out of the six conditions). *Moritella* spp. has been found in marine fish, seawater, and marine sediments [40,56]. This genus includes a well-established fish pathogen, *Moritella viscosa* [56], but almost no information is available regarding its potential role in fish spoilage. Our results indicate that further work needs to be carried out in order to elucidate its potential role as a spoiler microorganism, given its ability to compete with other microorganisms (demonstrated herein) and its close relationship with the genus *Shewanella* [57].

## 5. Conclusions

Our results strongly suggest that *Photobacterium* would be the major SSO of hake fillets stored under MAP (50% CO_2_/50% N_2_) in the range of temperatures between 1 and 7 °C. Further work will be required to elucidate if other microorganisms, such as *Psychrobacter, Moritella*, or *Carnobacterium*, also contribute to hake spoilage when stored under MAP.

## Figures and Tables

**Table 1:** Quality index method (QIM) attributes evaluated for hake fillets.

Quality Description	Scoring Description	Points
Muscle firmness	Very Firm (very defined myotomes)	0
	Firm (defined myotomes)	1
	Friable (moderately defined myotomes)	2
	Very friable (slightly defined myotomes)	3
Smell	Fresh, marine, seaweed	0
	Neutral, fruity	1
	Slightly sour, metallic or as preserved in the refrigerator	2
	Strong sour smelling, metallic or as preserved in refrigerator	3
Dryness (in the cutting surface)	Low	0
	Moderate	1
	High	2
	Very high yellowish, milky areas	3
Presence of marks or bruises	None	0
	Some	1
	Many	2
Skin (Ventral Zone)	Metallic and shiny, gray tones	0
	Slightly off-white	1
	Very White, brightless	2
Elasticity of the skin (pressing with several fingers and stretching outwards)	No fingerprint after pressing	0
	With fingerprint after pressing	1
**QIM SCORE**		0–14

**Table 2:** Recovery conditions for the different microbial groups investigated.

Microbial Group	Agar	Temp	Time	Atmosphere	Plating
**Aerobic Psychrotrophes**	LH Agar ^1^	7 °C	10 d	Aerobic	Spread
**Anaerobic Psychrotrophes**	LH Agar	7 °C	10–12 d	Anaerobic	Spread
***Pseudomonas***	GSP Agar ^2^	25 °C	24–48 h	Aerobic	Spread
***Shewanella***	Iron Agar ^3^	25 °C	3–4 d	Aerobic	Spread
**Lactic Acid Bacteria**	Elliker Agar ^4^	25 °C	24–48 h	Anaerobic^5^	Pour

^1^ Long and Hammer Agar (Broekaert et al., 2011 [22]). ^2^ Glutamate Starch Phenol Red Agar (Sigma-Aldrich, Steinheim, Germany) + Penicillin G (Sigma-Aldrich). ^3^ Iron Agar (Lingby) (Conda, Madrid, Spain). ^4^ Elliker Broth (Sigma-Aldrich) + Bacteriological Agar (Oxoid Basingstoke Hants, UK). ^5^ Anaerogen (Oxoid).

**Table 3:** Primers used for amplification of the different microbial groups investigated.

Microbial Group	Forward	Reverse	Source
***Photobacterium***	TACTGTTGAAGTGGCGAT	TCTGCTGGGCTTTCTAAT	This work
***Pseudomonas***	AAGCTAGAGTATGGTAGAG	CACCTCAGTGTCAGTAT	This work
***Shewanella***	GTAGGGAGGAAAGGTAATA	CTTTACGCCCAGTAATTC	This work

**Table 4:** Mean quantitation cycle (Cq) values (calculated from three technical replicates) for *Photobacterium* spp., *Shewanella* spp., and *Pseudomonas spp.* determined for each of the three independent samples (biological replicates) analyzed at the time of spoilage of hake fillets stored under MAP at the three studied temperatures. Data correspond to samples of batch A. The table also includes the standard deviation of the means.

		Temperature (°C)								
		1			4			7		
Microorganism	Sample/Replicate	1	2	3	1	2	3	1	2	3
*Photobacterium* spp.	**Cq**	22.53	22.36	22.12	23.08	22.86	23.23	22.27	21.90	21.97
	**S.D.**	0.597	0.487	0.327	0.433	0.333	0.321	0.342	0.299	0.552
*Shewanella* spp.	**Cq**	28.46	30.58	27.21	29.14	29.75	28.69	26.51	28.14	27.95
	**S.D.**	0.575	0.092	1.068	0.932	1.036	0.485	0.687	0.538	1.981
*Pseudomonas* spp.	**Cq**	33.90	34.16	29.19	31.88	32.97	31.06	29.22	31.68	31.69
	**S.D.**	0.233	0.010	0.766	0.555	1.503	1.615	1.873	2.140	2.071

## References

[1] Alheit J., Pitcher T.J. (1995). Hake: Biology, Fisheries and Markets.

[2] FAO The State of World Fisheries and Aquaculture 2016. http://www.fao.org/3/a-i5555e.pdf.

[3] Baixas-Nogueras S., Bover-Cid S., Veciana-Nogués M.T., Vidal-Carou M.C. (2003). Amino Acid-Decarboxylase activity in bacteria associated with Mediterranean hake spoilage. Eur. Food Res. Technol..

[4] MAPAMA (2016). El Mercado de la Merluza en España. https://www.mapa.gob.es/es/pesca/temas/mercados-economia-pesquera/informemerluzaabril2016_tcm30-291641.pdf.

[5] Dalgaard P. (1995). Qualitative and quantitative characterization of spoilage bacteria from packed fish. Int. J. Food Microbiol..

[6] Koutsoumanis K. (2001). Predictive modeling of the shelf life of fish under nonisothermal conditions. Appl. Environ. Microbiol..

[7] Amann R.I. (1995). Fluorescently labelled, rRNA-Targeted oligonucleotide probes in the study of microbial ecology. Mol. Ecol..

[8] Di Bella J.M., Bao Y., Gloor G.B., Burton J.P., Reid G. (2013). High throughput sequencing methods and analysis for microbiome research. J. Microbiol. Methods.

[9] Odeyemi O.A., Burke C.M., Bolch C.C.J., Stanley R. (2018). Seafood spoilage microbiota and associated volatile organic compounds at different storage temperatures and packaging conditions. Int. J. Food Microbiol..

[10] Parlapani F.F., Michailidou S., Anagnostopoulos D.A., Sakellariou A.K., Pasentsis K., Psomopoulos F., Argiriou A., Haroutounian S.A., Boziaris I.S. (2018). Microbial spoilage investigation of thawed common cuttlefish (*Sepia officinalis*) stored at 2 °C using next generation sequencing and volatilome analysis. Food Microbiol..

[11] Jia S., Huang Z., Lei Y., Zhang L., Li Y., Luo Y. (2018). Application of Illumina-MiSeq high throughput sequencing and culture-dependent techniques for the identification of microbiota of silver carp (*Hypophthalmichthys molitrix*) treated by tea polyphenols. Food Microbiol..

[12] Zotta T., Parente E., Ianniello R.G., De Filippis F., Ricciardi A. (2019). Dynamics of bacterial communities and interaction networks in thawed fish fillets during chilled storage in air. Int. J. Food Microbiol..

[13] Parlapani F.F., Michailidou S., Pasentsis K., Argiriou A., Krey G., Boziaris I.S. (2018). A Meta-Barcoding approach to assess and compare the storage Temperature-Dependent bacterial diversity of Gilt-Head sea bream (*Sparus aurata*) originating from fish farms from two geographically distinct areas of Greece. Int. J. Food Microbiol..

[14] Kuuliala L., Al Hage Y., Ioannidis A.-G., Sader M., Kerckhof F.-M., Vanderroost M., Boon N., De Baets B., De Meulenaer B., Ragaert P. (2018). Microbiological, chemical and sensory spoilage analysis of raw Atlantic cod (*Gadus morhua*) stored under modified atmospheres. Food Microbiol..

[15] Janda J.M., Abbott S.L. (2007). 16S rRNA gene sequencing for bacterial identification in the diagnostic laboratory: Pluses, perils, and pitfalls. J. Clin. Microbiol..

[16] Ercolini D. (2013). High-Throughput sequencing and metagenomics: Moving forward in the Culture-Independent analysis of food microbial ecology. Appl. Environ. Microbiol..

[17] Rodrigues P.A., Ferrari R.G., Conte-Junior C.A. (2018). Application of molecular tools to elucidate the microbiota of seafood. J. Appl. Microbiol..

[18] ISO 8586: 2012 (2012). Sensory Analysis—General Guidelines for the Selection, Training and Monitoring of Selected Assessors and Expert Sensory Assessors.

[19] Seafish (2010). Sensory Assessment Scoresheets for Fish and Shellfish-Torry & QIM. https://www.seafish.org/media/Publications/sensory_assessment_scoresheets_14_5_10.pdf.

[20] Woyewoda A.D., Shaw S.J., Ke P.J., Burns B.G. (1996). Recommended Laboratory Methods for Assessment of Fish Quality.

[21] Antunes-Rohling A., Artaiz A., Calero S., Halaihel N., Guillén S., Raso J., Álvarez I., Cebrián G. (2019). Modelling microbial growth in Modified-Atmosphere-Packed hake (*Merluccius merluccius*) fillets stored at different temperatures. Food Res. Int..

[22] Wilson B., Danilowicz B.S., Meijer W.G. (2008). The diversity of bacterial communities associated with Atlantic Cod *Gadus morhua*. Microb. Ecol..

[23] Klindworth A., Pruesse E., Schweer T., Peplies J., Quast C., Horn M., Glöckner F.O. (2013). Evaluation of general 16S ribosomal RNA gene PCR primers for classical and next-generation Sequencing-Based diversity studies. Nucleic Acids Res..

[24] Caporaso J.G., Kuczynski J., Stombaugh J., Bittinger K., Bushman F.D., Costello E.K., Fierer N., Peña A.G., Goodrich J.K., Gordon J.I. (2010). QIIME allows analysis of High-Throughput community sequencing data. Nat. Methods.

[25] Callahan B.J., McMurdie P.J., Rosen M.J., Han A.W., Johnson A.J., Holmes S.P. (2016). DADA2: High-Resolution sample inference from Illumina amplicon data. Nat. Methods.

[26] Baixas-Nogueras S., Bover-Cid S., Veciana-Nogués T., Nunes M.L., Vidal-Carou M.C. (2003). Development of a quality index method to evaluate freshness in Mediterranean hake (*Merluccius merluccius*). J. Food Sci..

[27] Gillespie N.C., MacRae I.C. (1975). The bacterial flora of some Queensland fish and its ability to cause spoilage. J. Appl. Bacteriol..

[28] Shewan J.M., Murray C.K., Russell A.D., Fuller R. (1979). The microbial spoilage of fish with special reference to the role of psychrophiles. Cold Tolerant Microbes in Spoilage and the Environment.

[29] Gram L., Dalgaard P. (2002). Fish spoilage Bacteria-Problems and solutions. Curr. Opin. Biotechnol..

[30] Gram L., Ravn L., Rasch M., Bruhn J.B., Christensen A.B., Givskov M. (2002). Food spoilage—Interactions between food spoilage bacteria. Int. J. Food Microbiol..

[31] Hilton S.K., Castro-Nallar E., Pérez-Losada M., Toma I., McCaffrey T.A., Hoffman E.P., Siegel M.O., Simon G.L., Evan Johnson W., Crandall K.A. (2016). Metataxonomic and metagenomic approaches vs. Culture-Based techniques for clinical pathology. Front. Microbiol..

[32] Wang Y., Hammes F., De Roy K., Verstraete W., Boon N. (2010). Past, present and future applications of flow cytometry in aquatic microbiology. Trends Biotechnol..

[33] Didelot X., Bowden R., Wilson D.J., Peto T.E., Crook D.W. (2012). Transforming clinical microbiology with bacterial genome sequencing. Nat. Rev. Genet..

[34] McLoughlin K.S. (2011). Microarrays for pathogen detection and analysis. Brief. Funct. Genom..

[35] Metzker M.L. (2009). Sequencing technologies—The next generation. Nat. Rev. Genet..

[36] Adams I.P., Glover R.H., Monger W.A., Mumford R., Jackeviciene E., Navalinskiene M., Samuitiene M., Boonham N. (2009). Next-Generation sequencing and metagenomic analysis: A universal diagnostic tool in plant virology. Mol. Plant Pathol..

[37] Dunne W., Westblade L., Ford B. (2012). Next-Generation and Whole-Genome sequencing in the diagnostic clinical microbiology laboratory. Eur. J. Clin. Microbiol. Infect. Dis..

[38] Boziaris I.S., Parlapani F.F., Bevilacqua A., Corbo M.R., Sinigaglia M. (2016). Chapter 3: Specific Spoilage Organisms (SSO) in Fish. Microbiological Quality of Food: Foodborne Spoilers.

[39] Macé S., Cornet J., Chevalier F., Cardinal M., Pilet M.F., Dousset X., Joffraud J.J. (2012). Characterization of the spoilage microbiota in raw salmon (*Salmo salar*) steaks stored under vacuum or modified atmosphere packaging combining conventional methods and PCR–TTGE. Food Microbiol..

[40] Reynisson E., Lauzon H.L., Magnússon H., Jónsdóttir R., Ólafsdóttir G., Marteinsson V., Hreggvidsson G. (2009). Bacterial composition and succession during storage of North-Atlantic cod (*Gadus morhua*) at superchilled temperatures. BMC Microbiol..

[41] Dalgaard P., Mejlholm O., Christiansen T.J., Huss H.H. (1997). Importance of *Photobacterium phosphoreum* in relation to spoilage of modified Atmosphere-Packed fish products. Lett. Appl. Microbiol..

[42] Rudi K., Maugesten T., Hannevik S.E., Nissen H. (2004). Explorative multivariate analyses of 16S rRNA gene data from microbial communities in Modified-Atmosphere-Packed salmon and coalfish. Appl. Environ. Microbiol..

[43] Hovda M.B., Lunestad B.T., Sivertsvik M., Rosnes J.T. (2007). Characterisation of the bacterial flora of modified atmosphere packaged farmed Atlantic cod (*Gadus morhua*) by PCR-DGGE of conserved 16S rRNA gene regions). Int. J. Food Microbiol..

[44] Shahidi F., Jones Y., Kitts D.D. (1997). Seafood Safety, Processing and Biotechnology.

[45] Bowman J.P., Dworkin M., Falkow S., Rosenberg E., Schleifer K.-H., Stackebrandt E. (2006). The genus *Psychrobacter*. The Prokaryotes—A Handbook on the Biology of Bacteria.

[46] Bjorkevoll I., Olsen R.L., Skjerdal O.T. (2003). Origin and spoilage potential of the microbiota dominating genus *Psychrobacter* in sterile rehydrated Salt-Cured and dried salt-cured cod (Gadus morhua). Int. J. Food Microbiol..

[47] Broekaert K., Heyndrickx M., Herman L., Devlieghere F., Vlaemynck G. (2011). Seafood quality analysis: Molecular identification of dominant microbiota after ice storage on several growth media. Food Microbiol..

[48] Romero J., Gonzalez N., Espejo R.T. (2002). Marine *Pseudoalteromonas* sp. composes most of the bacterial population developed in oysters (*Tiostrea chilensis*) spoiled during storage. J. Food Sci..

[49] Mejlholm O., Boknaes N., Dalgaard P. (2005). Shelf life and safety aspects of chilled cooked and peeled shrimps (*Pandalus borealis*) in modified atmosphere packaging. J. Appl. Microbiol..

[50] Prapaiwong N., Wallace R.K., Arias C.R. (2009). Bacterial loads and microbial composition in high pressure treated oysters during storage. Int. J. Food Microbiol..

[51] Rodriguez-Calleja J.M., Patterson M.F., Garcia-Lopez I., Santos J.A., Otero A., Garcia-Lopez M.L. (2005). Incidence, radioresistance, and behavior of *Psychrobacter* spp. in rabbit meat. J. Food Prot..

[52] Gennari M., Parini M., Volpon D., Serio M. (1992). Isolation and characterisation by conventional methods and genetic transformation of *Psychrobacter* and *Acinetobacter* from fresh and spoiled meat, milk and cheese. Int. J. Food Microbiol..

[53] Broekaert K., Noseda B., Heyndrickx M., Vlaemynck G., Devlieghere F. (2013). Volatile compounds associated with *Psychrobacter* spp. and *Pseudoalteromonas* spp., the dominant microbiota of brown shrimp (Crangon crangon) during aerobic storage. Int. J. Food Microbiol..

[54] Leisner J.J., Laursen B.G., Prévost H., Drider D., Dalgaard P. (2007). *Carnobacterium:* Positive and negative effects in the environment and in foods. FEMS Microbiol. Rev..

[55] Joffraud J.J. (2006). Effect of bacterial interactions on the spoilage of cold-smoked salmon. Int J. Food Microbiol..

[56] Urakawa H., Kita-Tsukamoto K., Steven S.E., Ohwada K., Colwell R.R. (1998). A proposal to transfer *Vibrio marinus* (Russell 1891) to a new genus *Moritella gen. nov*. as *Moritella marina comb. nov*. FEMS Microbiol. Lett..

[57] Benediktsdoóttir E., Heiðarsdoóttir K.J. (2007). Growth and lysis of the fish pathogen *Moritella viscosa*. Lett. Appl. Microbiol..

